# Does the Risk of Ovarian Malignancy Algorithm Provide Better Diagnostic Performance Than HE4 and CA125 in the Presurgical Differentiation of Adnexal Tumors in Polish Women?

**DOI:** 10.1155/2018/5289804

**Published:** 2018-04-10

**Authors:** Nabil Abdalla, Robert Piorkowski, Michal Bachanek, Pawel Stanirowski, Krzysztof Cendrowski, Wlodzimierz Sawicki

**Affiliations:** Department of Obstetrics, Gynecology and Oncology, Second Faculty of Medicine, Medical University of Warsaw, Warsaw, Poland

## Abstract

**Aim:**

This study compared the diagnostic performance of the Risk of Ovarian Malignancy Algorithm (ROMA) and HE4 and CA125 for the presurgical differentiation of adnexal tumors.

**Material and Methods:**

This prospective study included 302 patients admitted for surgical treatment due to adnexal tumors. The ROMA was calculated depending on CA125, HE4, and menopausal status.

**Results:**

Fifty patients were diagnosed with malignant disease. In the differentiation of malignant from nonmalignant adnexal tumors, the area under curve (AUC) was higher for ROMA and HE4 than that for CA125 in both the premenopausal and postmenopausal subgroups. In the differentiation of stage I FIGO malignancies and epithelial ovarian cancer from nonmalignant pathologies, the AUC of HE4 and ROMA was higher than that of CA125. The ROMA performed significantly better than CA125 in the differentiation of all malignancies and differentiation of stage I FIGO malignancies from nonmalignant pathologies (*p* = 0.043 and *p* = 0.025, resp.). There were no significant differences between the ROMA and the tumor markers for any other variants.

**Conclusions:**

The ROMA is more useful than CA125 for the differentiation of malignant (including stage I FIGO) from nonmalignant adnexal tumors. It is also as useful as HE4 and CA125 for the differentiation of epithelial ovarian cancer from nonmalignant adnexal tumors.

## 1. Introduction

Adnexal tumors represent a wide variety of diseases that may affect the ovaries and/or fallopian tubes. Tumors of the adjacent structures, such as uterine fibroids, can mimic adnexal tumors. Ovarian tumors can be functional, benign, or malignant. Ovarian malignancies can be primary or secondary, with primary tumors originating from epithelial cells, sex cords, or germinal cells [1, 2]. The heterogeneous nature of adnexal masses is one of the causes of preoperative difficulties in these tumors [3, 4]. Ovarian cancer (OC) is the fifth most common malignancy among women (5% of all cancers) and the fourth most common cause of mortality related to malignancy in Poland [5].

The tumor marker CA125, initially described by Bast et al., is widely used for the routine diagnosis of adnexal masses [6]. It is also used for monitoring the response to treatment, follow-up of the disease, and detection of disease recurrence [7]. This tumor marker can be increased in several gynecological and nongynecological diseases, and this reduces the diagnostic accuracy for the detection of ovarian cancer [8–11].

Endometriosis is a prominent cause of increased CA125 [12]. In 1991, Kirchoff et al. identified a major human epididymis-specific cDNA that encodes a protein with sequence homology to extracellular proteinase inhibitors. Northern blot and in situ transcript hybridization specifically localized the HE4 (human epididymis gene product) mRNA to the distal section of epithelial cells in the epididymal duct [13]. Subsequent studies have shown that HE4 is elevated in 90% of serous ovarian cancer cases and in most cases of endometrioid and clear cell cancer, whereas mucinous and germ cell tumors rarely express HE4 [14]. The marker HE4 is significantly increased in ovarian and endometrial cancer, but not in cases of endometriosis [12]; furthermore, it is less frequently elevated compared to CA125 in patients with benign disease, especially in premenopausal patients [15]. HE4 can be increased in nongynecological malignancies [16].

Several different mathematical models and scoring systems have been created, based on clinical features, ultrasound findings, and/or serum level of tumor markers, aimed at increasing the diagnostic performance of each individual parameter [3]. One such model is the Risk of Ovarian Malignancy Algorithm (ROMA) created by Moore et al. The ROMA combines the tumor markers CA125 and HE4 using two formulas, taking into account the menopausal status of each patient. The ROMA can classify patients as being at low and high risks for epithelial ovarian cancer (EOC), and 93.8% of cases in Moore et al.'s study were correctly classified under the high-risk category [17]. In 2010, Moore et al. concluded that ROMA achieved higher sensitivity than the risk of malignancy index (RMI) for identifying EOC in a prospective multicenter trial in 457 patients. The authors suggested that radiological imaging studies without central review may more accurately reflect actual clinical practice. By contrast, the serum levels of tumor markers provide objective results that showed more utility and more consistency and reproducibility between centers and between regions [18]. The analysis by Nolen et al. reaffirmed the superiority of assessing a combination of HE4/CA125 for the diagnosis of OC [19]. In 2011, the use of ROMA was validated in a low-risk population of women with adnexal masses who presented to a general practitioner. Despite the low incidence of malignancies in this trial (15% of all cases and 10% for EOC), the ROMA stratified patients into high- and low-risk groups, with 93.8% sensitivity and 74.9% specificity for predicting OC [20].

A meta-analysis by Li et al. in 2012 analyzed the performance of HE4, CA125, and ROMA in 11 studies and data from 7792 tests. The authors concluded that HE4 was no better than CA125 for either EOC or OC prediction, whereas ROMA was a promising predictor of EOC that could replace CA125. The overall estimates of ROMA for EOC prediction were as follows: a sensitivity of 89% (95% CI: 84%–93%), specificity of 83% (95% CI: 77%–88%), and AUC of 0.93 (95% CI: 0.90–0.95). However, the authors concluded that ROMA utilization requires further evaluation [21].

A meta-analysis by Dayyani et al. in 2016 analyzed five studies incorporating 1975 patients with adnexal masses. On the basis of the AUC (95% confidence interval) data for all patients, the authors concluded that the ROMA (0.921 [0.855–0.960]) showed a numerically greater diagnostic performance than CA125 (0.883 [0.771–0.950]) and HE4 (0.899 [0.835–0.943]). Similar results were shown in each of the subgroup populations, in particular, postmenopausal patients and patients with early OC. The meta-analysis had strict selection criteria for inclusion [22]. Several studies compared ROMA with CA125 and HE4 with contradicting results, which can be attributed to the subgroups analyzed, populations studied, oncology profile of the investigating center, cut-off levels of diagnostic tests, and choice of certain ovarian pathologies [23–26].

The optimum diagnosis of the malignant status of masses is important as it facilitates the selection of patients with malignant masses who need urgent referral to gynecological oncology centers and consequently improves the overall survival rate for patients with ovarian cancer [27].

The aim of the study was to compare the diagnostic performance of ROMA with tumor markers HE4 and CA125 in a selected Polish population. The comparison was performed for all patients, as well as for premenopausal and postmenopausal subgroups. In addition, the diagnostic value of ROMA was compared with that of tumor markers for differentiating between malignant adnexal tumor stage I according to FIGO and nonmalignant adnexal tumors and the differentiation of EOC from other nonmalignant adnexal tumors.

## 2. Material and Methods

This was a prospective study of 302 patients with adnexal masses referred to our clinic for surgery between October 2012 and April 2015. Patients were referred from physicians with different experience levels. The referral of patients was considered at outpatient units according to the local recommendations of these units depending on history, clinical examination, tumor markers, and ultrasound examination. The following inclusion criteria were used: age older than 18 years, measurement of serum concentration of tumor markers CA125 and HE4 less than five days before surgical intervention, histopathological results for the adnexal lesion, and obtainment of consent. Exclusion criteria included pregnancy, renal diseases, history of malignancy, chemotherapy and/or radiotherapy, fibroids > 5 cm, and a lack of histological assessment of the adnexal tumor. Serum HE4 and CA125 levels were measured for each patient at the same time with the same apparatus (Cobas 8000-e602), using an electrochemiluminescence immunoassay. The two logistic regression formulas described by Moore et al. were used to calculate ROMA. These formulas include a natural logarithm (ln) of CA125 and HE4 values. The predictive index (PI) was calculated for the premenopausal and postmenopausal subgroups. For the premenopausal subgroup, the following formula was used: predictive index (PI) = −12.0 + 2.38^∗^ln(HE4) + 0.0626^∗^ln(CA125). The formula for the postmenopausal subgroup was as follows: predictive index (PI) = −8.09 + 1.04^∗^ln(HE4) + 0.732^∗^ln(CA125). The following formula was applied to calculate the risk of malignancy based on the ROMA (%): (%) = exp(PI)/[1/exp(PI)]∗100 [17]. The cut-off level recommended by the manufacturer for CA125 was 35 U/mL, while the cut-off level for HE4 was 70 and 140 pmol/L for premenopausal and postmenopausal patients, respectively. The ROMA cut-off levels for high-risk patients were 11.4% and 29.9% for premenopausal and postmenopausal patients, respectively. The final decision for surgery was made individually by at least two gynecologists depending on the classical risk of malignancy index, tumor markers levels, and subjective assessment of adnexal tumors considering the patient's preference. The definitive diagnosis of the adnexal mass was established by the histopathological examination of the adnexal mass. Borderline tumors were considered as malignant in the statistical analysis. Malignant masses were staged according to the International Federation of Gynecology and Obstetrics (FIGO) guidelines. Menopause was defined as at least one year of absence of menstruation [17]. Descriptive analysis was used for patients with different adnexal pathologies. The Mann–Whitney *U* test was used to assess the statistical difference between mean serum levels of HE4 and CA125. The sensitivity, specificity, positive and negative predictive values, and accuracy of tumor markers and ROMA were calculated to distinguish different adnexal pathologies among different groups of patients. The receiver operating characteristics area under the curve (ROC-AUC) was constructed for each diagnostic test. The AUC of these tests were compared to each other using Hanley and McNeil methods. A *p* value less than 0.05 was assumed to be statistically significant. The study protocol was approved by the local ethical committee (Nr KB/192/2012).

## 3. Results

A total of 302 patients were included in the study. The patients were aged 18–85 years with a mean of 48.7 years and a standard deviation (SD) of 16.79 years. Premenopausal patients comprised the majority of patients (*n* = 188 [62.3%]) and only 114 (37.7%) patients were postmenopausal. Final histopathological examinations revealed 252 (83.4%) cases of nonmalignant and 50 (16.6%) cases of malignant adnexal pathologies. The vast majority (*n* = 48 [96%]) of malignant tumors were of ovarian origin; two were fallopian tube malignancies. The number of patients with each FIGO stage of malignant ovarian pathologies was as follows: stage IA, 9; IC, 6; IIA, 4; IIC, 1; IIIA, 1; IIIB, 2; IIIC, 24; and IVB, 1. Most malignant ovarian cases were of epithelial origin (*n* = 46). Tubal malignancies included one case of stage IC and one of IIIA. The distribution of final histological diagnoses of adnexal masses is presented in Table 1.

The serum level of HE4 and CA125 among the whole group, the premenopausal subgroup, and postmenopausal subgroup is presented in Table 2. The Mann–Whitney *U* test showed that both tumor markers showed significantly higher serum levels in patients with malignant adnexal masses.

The sensitivity, specificity, positive and negative predictive values, and the accuracy of HE4, CA125, and ROMA considering menopausal status are presented in Table 3. The diagnostic performance of these tests for differentiation of stage I FIGO malignant adnexal tumors and EOC from nonmalignant adnexal tumors is displayed in Table 4.

A ROC-AUC was computed for tumor markers and ROMA for the whole group, as well as for the premenopausal and postmenopausal subgroups. All diagnostic tests significantly differentiated malignant adnexal tumors from nonmalignant adnexal tumors in the analysis of the whole group, as well as analysis of the postmenopausal and premenopausal subgroups. A ROC-AUC was also constructed for the tumor markers and ROMA to assess their performance for differentiation between stage I FIGO malignant adnexal tumors and EOC from nonmalignant adnexal tumors. Both HE4 and ROMA were significantly better than CA125 in differentiating stage I FIGO malignant tumors from nonmalignant adnexal tumors. Both tumor markers and ROMA were able to differentiate epithelial ovarian cancer from nonmalignant adnexal tumors.

**Figure 1:**
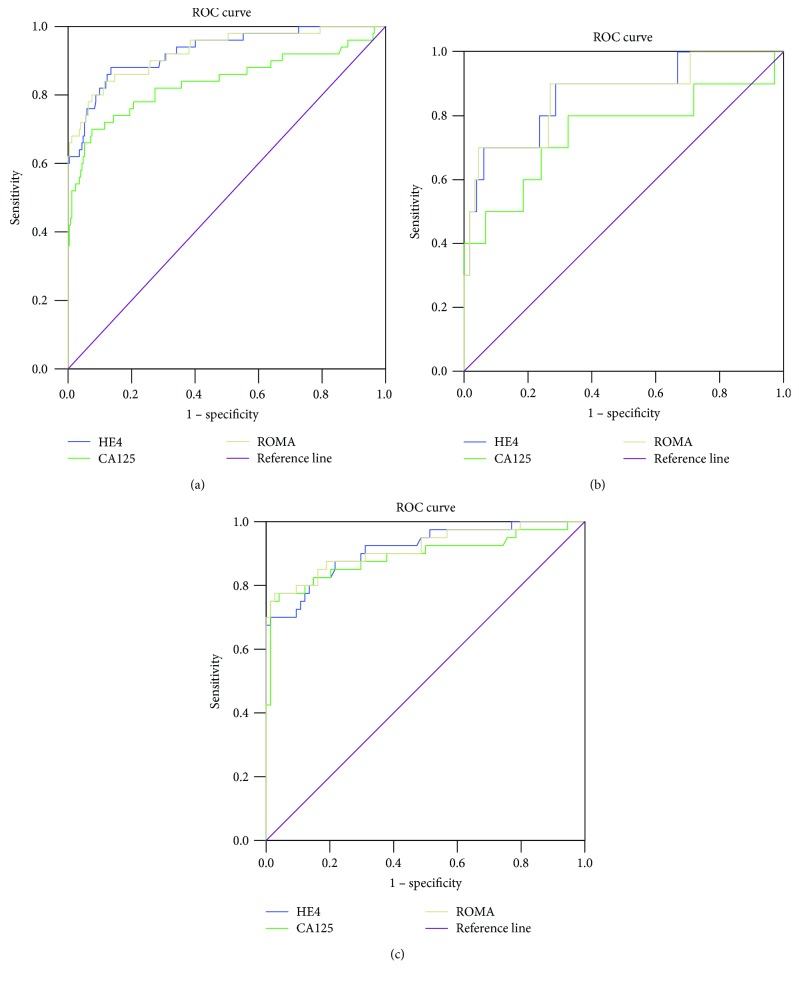
The ROC-AUC for HE4, CA125, and ROMA for the differentiation between malignant and nonmalignant adnexal masses in the whole group (a), premenopausal subgroup (b), and postmenopausal subgroup (c).

**Figure 2:**
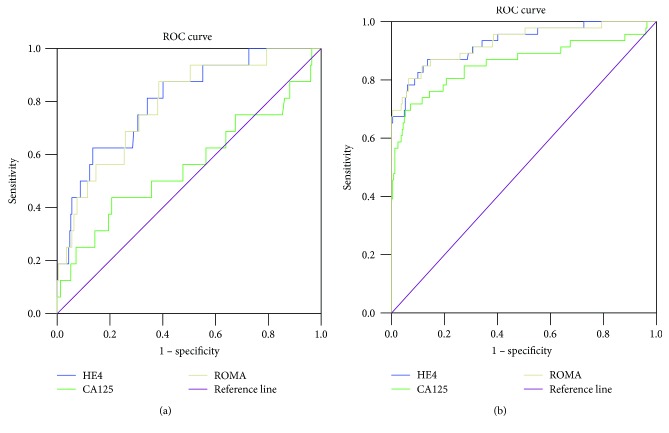
(a) The ROC-AUC of HE4, CA125, and ROMA for the differentiation of stage I FIGO malignant tumors from nonmalignant adnexal tumors. FIGO: Fédération Internationale de Gynécologie et d'Obstétrique. (b) The ROC-AUC of HE4, CA125, and ROMA for the differentiation of epithelial ovarian cancer from nonmalignant adnexal tumors.

The HE4, CA125, and ROMA AUCs, as well as the statistical differences and optimal cut-offs for the whole group and the premenopausal and postmenopausal subgroups are presented in Table 5. The tumor markers and ROMA AUCs for the differentiation of stage I FIGO malignant adnexal tumors and EOC from nonmalignant adnexal tumors are shown in Table 6. The ROC-AUCs for HE4, CA125, and ROMA for the whole group, the premenopausal subgroup, and the postmenopausal subgroup are presented in Figures 1(a)–1(c), respectively. The ROC-AUCs for HE4, CA125, and ROMA for the differentiation of stage I FIGO malignant tumors from nonmalignant adnexal tumors are shown in Figure 2(a). The ROC-AUCs of these tests for the differentiation of EOC from nonmalignant adnexal tumors are shown in Figure 2(b).

The AUCs of the diagnostic tests were compared using the Hanley and McNeil test. The results are presented in Table 7. The ROMA and HE4 were significantly (although marginally) better than CA125 for the differentiation of malignant tumors from nonmalignant tumors in the whole group (*p* = 0.043 and *p* = 0.043, resp.). Similarly, HE4 and ROMA were significantly better than CA125 for the differentiation of stage I FIGO malignant tumors from nonmalignant adnexal masses. The ROMA was not significantly better than HE4 or CA125 for the differentiation of EOC from nonmalignant adnexal masses in the whole group.

## 4. Discussion

Our study demonstrated that ROMA had the best ROC-AUC for the differentiation of EOC from nonmalignant adnexal masses, while CA125 had the worst ROC-AUC. However, there were no significant statistical differences in the performance of HE4, CA125, and ROMA. Our results are supported by those of Terlikowska et al., who also found no statistically significant difference between the ROC-AUC of these diagnostic tests [28]. The reported diagnostic performance of tumor markers and ROMA varies widely in the literature. Cho et al. reported that ROMA and HE4 showed significantly better performance than CA125 [29]. Romagnolo et al. reported that for the differentiation of EOC from benign adnexal diseases, the ROMA had the highest ROC-AUC in both premenopausal and postmenopausal patients [30]. Shen et al. revealed that the ROC-AUC of ROMA was significantly higher than that of HE4 and CA125 for the differentiation of all malignant diseases (including EOC, borderline tumors, and metastatic tumors) from other benign diseases. The high specificity and positive predictive value of HE4 may decrease the usefulness of adding CA125 into the diagnostic protocol, as patients with elevated HE4 are already considered to be at high risk [31]. By contrast, Van Gorp et al. revealed an insignificant difference in the diagnostic performance of HE4 and ROMA compared to CA125 in the differentiation of all malignant from nonmalignant pelvic masses in a prospective study of 389 patients [32]. Jacob et al. showed that the combination of both markers does not improve the diagnostic performance compared to HE4 alone and does not overcome the inability of both markers to adequately detect early-stage epithelial ovarian cancers. The authors suggested that the combination of HE4 and CA125 is beneficial in patients with a high score on the RMI due to elevated CA125. In that case, a normal level of HE4 will infer endometriosis rather than OC [33]. Fujiwara et al. analyzed the role of tumor markers and ROMA as diagnostic tools for type I and II epithelial ovarian cancers. For type I, HE4 and ROMA showed better sensitivity than CA125. At 75% specificity, the sensitivities of CA125 and HE4 were 92.1% for both markers for type II and 51.5% and 78.8% for type I, respectively. The sensitivity of the ROMA was better than the sensitivities of CA125 and HE4 and reached 84.8% and 97.4% for type I and type II, respectively [34]. In our study, when considering the total patient population and the premenopausal subgroup, CA125 had the highest sensitivity. In the postmenopausal subgroup, CA125 and ROMA had similar sensitivities. Compared to that of CA125 and that of ROMA, HE4 had the highest specificity for the whole group and for the premenopausal and postmenopausal subgroups. All diagnostic tests successfully differentiated adnexal masses, irrespective of menopausal status. For the whole group, HE4 and ROMA had the highest ROC-AUC, while HE4 had the highest ROC-AUC for the premenopausal subgroup; additionally, ROMA had the highest ROC-AUC for the postmenopausal subgroup. In the whole group, HE4 and ROMA were significantly (although marginally) superior to CA125 for the presurgical differentiation of adnexal masses (*p* = 0.043).

Age has a strong effect on serum levels of HE4. Urban et al. concluded that thresholds for HE4 are best defined for women of specific ages. Age-specific population thresholds for HE4 at 95% specificity ranged from 41.4 pmol/L for women aged 30 to 82.1 pmol/L for women aged 80 years [35]. Chudecka-Glaz et al. suggested a modified ROMA algorithm using a specific age range instead of the dichotomization of patients according to pre- and postmenopausal status. The authors concluded that the modified ROMA had higher specificity and positive predictive value than the original ROMA and suggested that a single cut-off level may be obtained for the entire population, regardless of menopausal status [36].

The positive predictive value of tumor markers and ROMA was much lower in the premenopausal subgroup compared to the postmenopausal subgroup. This difference may be attributed to a higher proportion (80%) of malignant cases among postmenopausal patients and the presence of endometriosis in premenopausal patients. Endometriosis is a main factor that may falsely increase serum levels of CA125 [37].

The consideration of tumor markers and ROMA for clinic-surgical assessment was beyond the scope of our study. Bandiera et al. revealed that in patients with EOC, elevated tumor marker levels and ROMA were associated with advanced FIGO stage, suboptimal debulking, ascites, positive cytology, lymph node involvement, and advanced age. The authors' multivariable analysis showed that HE4 and ROMA were independent prognostic factors for shorter overall survival rate, disease-free survival rate, and progression-free survival rate [9]. Li et al. demonstrated that high ROMA scores correlated with advanced ovarian cancer and ROMA were the strongest predictor of FIGO stage, with the highest specificity, accuracy, and positive predictive value (84.4%, 82.5%, and 87.0% for postmenopausal patients, resp., and 89.3%, 85.6%, and 74.3% for premenopausal patients, resp.) [38].

The BRCA1 mutation is a risk factor for ovarian cancer. In patients with BRCA1 mutation, the role of ROMA seems important. Chudecka-Glaz et al. investigated the diagnostic performance of tumor markers and ROMA in differentiation of pelvic masses, taking into consideration the BRCA1 mutation. In comparing ovarian cancer with benign ovarian disease in patients with BRCA1 mutation, ROMA had the best ROC-AUC, followed by CA125 and then by HE4. The authors showed that ROMA significantly differed from HE4 for the diagnosis. Similar results were revealed in postmenopausal patients. In premenopausal patients, the results were different in that CA125 had the best ROC-AUC followed by ROMA and then by HE4. However, there was no significant statistical difference in the diagnostic performance of these tests in this group of patients [39]. Chudecka-Glaz et al., in another study, concluded that patients with BRCA1 gene mutations have relatively low serum HE4 levels. Even the slightest elevation in HE4 or CA125 levels in female BRCA1 carriers undergoing prophylactic surgery should significantly increase oncological alertness [40].

The strength of our study is its prospective nature defined by a strict protocol. The tumor markers were measured within five days before surgical intervention and measured in the same way throughout the study. However, our study was not without limitations. It may have been affected by certain factors which should be considered in the interpretation of the results. The prevalence of malignancy was much higher than the prevalence in the community due to the referral of adnexal tumors suspicious of malignancy to our mixed gynecology-oncology referral center. However, the overall number of malignant cases in our study was lower than the number of nonmalignant cases. In consequence, our results cannot be applied to the primary healthcare setting. Simultaneously, because of the lower number of malignant adnexal tumors compared to that of nonmalignant tumor, our results cannot be applied to purely oncological centers where the prevalence of malignant cases is higher. This study enrolled patients who were referred for surgical management. The referral was dependent on ultrasound scans, clinical features, and tumor marker levels. We were unable to predict the number of cases that could have been referred for treatment earlier. Similarly, we were unable to predict the number of patients who were missed at the primary diagnostic level before referral because they were instead diagnosed with ovarian functional changes. Missed diagnosis and/or delayed referral to oncological centers increase the number of cases with advanced malignancy. We adopted exclusion criteria similar to those found in the literature for comparing the results. However, these criteria excluded the most difficult cases of adnexal masses. Renal diseases elevate the HE4 level while the level among pregnant women is lower than that among premenopausal patients [41, 42]. The incorporation of borderline ovarian tumors into the malignant group may have had an effect on results, although the overall number of these tumors in our study was small. Anton et al. showed higher sensitivity of tumor markers and ROMA when borderline ovarian tumors were classified as low-risk tumors [25]. Braicu et al. concluded that both CA125 and HE4 were not reliable biomarkers for the diagnosis of borderline ovarian tumors or for predicting the presence of invasive implants [43].

One benefit of ROMA that may distinguish it from other diagnostic modalities for adnexal masses is the elimination of ultrasound, which is highly dependent on examiner experience. For this reason, the use of serum markers in the diagnostic approach can be more objective and comparable [44].

There is still considerable debate on whether an ultrasound-based model or a tumor marker-based model should be used. In one study, a multicenter external validation using decision-curve analysis was performed to assess the clinical utility of risk models to refer patients with adnexal masses to specialized oncology care and concluded that three International Ovarian Tumor Analysis (IOTA) group models, including the ADNEX model, logistic regression model (LR2), and simple rules are clinically more useful than RMI and ROMA to select patients with adnexal masses for specialized oncology care [45]. In our study, ROMA is more useful than CA125 for the differentiation of malignant (including stage I FIGO) from nonmalignant adnexal tumors indicating the feasible use of ROMA in the presurgical differentiation of adnexal tumors.

Further modification of the ROMA might be needed given the lack of highly specific and sensitive diagnostic tests for the diagnosis of malignant adnexal tumors. Jeong et al. proposed a new reporting strategy for interpreting ROMA values based on the analytical measurement range and qualified-intervals of the HE4 and CA125 results. Reporting algorithms for the ROMA value were classified into three main categories. In the first category, the numerical ROMA value can be reported when quantitative HE4 and CA125 levels are reliable. In the second and third categories, patients were considered as low risk or undetermined depending on the levels of HE4 or CA125. The authors concluded that the new reporting strategy will provide more information on the utility of ROMA values in clinical practice [46]. Molina et al. suggested that the best algorithm to predict ovarian cancer was to classify all patients with increased HE4 as high-risk patients and to use ROMA for patients with normal HE4 and increased CA125 serum levels [47]. Despite variations between diagnostic tools assessed in our study, all of them significantly differentiated malignant from nonmalignant tumors preoperatively. Results showed the superiority of the ROMA compared to CA125 to differentiate malignancies, including stage I FIGO, from nonmalignant tumors. By contrast, the results showed no significant statistical difference between the overall performance of ROMA and HE4 in the differentiation of malignant and nonmalignant cases.

Our results may have economic implications for daily practice where only HE4 can be ordered for patients with adnexal masses, as ROMA requires the measurement of both tumor markers. Currently, most cases of ovarian cancer are detected in the advanced stages before referral to oncological centers; therefore, there is a need to use diagnostic tools to identify ovarian cancer as early as possible. In our study, both HE4 and ROMA were more helpful for detecting cases of stage I FIGO among patients referred to the oncology center. The appropriate diagnosis of early-stage malignancies at the oncology center enables the choice of radical surgery. Simultaneously, the diagnosis of nonmalignant adnexal tumor enables physicians to choose more conservative surgical interventions.

## 5. Conclusions

In the whole group, ROMA and HE4 were more useful than CA125 for the differentiation of malignant from nonmalignant adnexal tumors. This is also true for the differentiation of stage I FIGO malignant tumors from nonmalignant adnexal tumors. The ROMA is not significantly superior to tumor markers for the differentiation of EOC from nonmalignant adnexal tumors. Further studies on the diagnostic performance of the ROMA are needed to confirm our results.

## Figures and Tables

**Table 1:** The distribution of final histological diagnoses of adnexal masses.

Main adnexal type	Histological subtype	*N* (%)
Nonmalignant *n* = 252	Endometriotic cyst	56 (22.2%)
	Dermoid cyst	54 (21.4%)
	Simple cyst	52 (20.6%)
	Serous cystadenoma	41 (16.3%)
	Mucinous cystadenoma	19 (7.5%)
	Tubo-ovarian abscess/salpingitis	16 (6.3%)
	Paraductal cyst	8 (3.2%)
	Ovarian fibroma	6 (2.4%)

Malignant *n* = 50	Ovarian serous tumor	22 (44%)
	Ovarian endometrioid tumor	11 (22%)
	Ovarian serous borderline	5 (10%)
	Ovarian mucinous tumors	3 (6%)
	Ovarian clear cell tumor	3 (6%)
	Ovarian mucinous borderline tumors	2 (4%)
	Fallopian tube malignancy	2 (4%)
	Ovarian folliculoma	1 (2%)
	Ovarian sarcoma	1 (2%)

**Table 2:** Difference in serum tumor markers among groups by the Mann–Whitney *U* test.

Group studied	Tumor marker	Mean serum tumor marker levels among nonmalignant adnexal masses	Mean serum tumor marker levels among malignant adnexal masses	*p* value
Whole group (*n* = 302)	HE4 (pmol/L)	53.3	1138.8	<0.001
	CA125 (U/mL)	41.8	588.3	<0.001
Premenopausal subgroup (*n* = 188)	HE4 (pmol/L)	46.6	673.6	<0.001
	CA125 (U/mL)	48.1	1138.8	0.008
Postmenopausal subgroup (*n* = 114)	HE4 (pmol/L)	69.3	1255.1	<0.001
	CA125 (U/mL)	26.5	450.7	<0.001

**Table 3:** The sensitivity, specificity, positive predictive value, negative predictive value, and diagnostic accuracy of H4, CA125, and ROMA according to menopausal status.

Group	Diagnostic test	Sensitivity (%) (95% CI)	Specificity (%) (95% CI)	Positive predictive value (%) (95% CI)	Negative predictive value (%) (95% CI)	Diagnostic accuracy (%) (95% CI)
Whole group (*n* = 302)	HE4	70% (61%–79%)	92.5% (89.2%–95.7%)	78.7% (70.1%–87.2%)	88.6% (84.8%–92.4%)	86.1% (82.5%–89.7%)
	CA125	82% (71.4%–92.6%)	68.3% (62.5%–74%)	33.9% (25.5%–42.3%)	95% (91.9%–98.2%)	70.5% (65.4%–75.7%)
	ROMA	80% (68.9%–91.1%)	82.5% (77.9%–87.2%)	47.6% (36.9%–58.3%)	95.4% (92.6%–98.2%)	82.1% (77.8%–86.4%)
Premenopausal subgroup (*n* = 188)	HE4	70% (41.6%–98.4%)	92.7% (88.9%–96.5%)	35% (14.1%–55.9%)	98.2% (96.2%–100%)	91.5% (87.5%–95.5%)
	CA125	80% (55.2%–100%)	62.4% (55.2%–69.5%)	10.7% (3.7%–17.7%)	98.2% (95.8%–100%)	63.3% (56.4%–70.2%)
	ROMA	70% (41.6%–98.4%)	82% (76.4%–87.7%)	17.9% (5.9%–30%)	98% (95.7%–100%)	81.4% (75.8%–86.9%)
Postmenopausal subgroup (*n* = 114)	HE4	70% (55.8%–84.2%)	91.9% (85.7%–98.1%)	82.4% (69.5%–95.2%)	85% (77.2%–92.8%)	84.2% (77.5%–90.9%)
	CA125	82.5% (70.7%–94.3%)	82.4% (73.8%–91.1%)	71.7% (58.7%–84.8%)	89.7% (82.5%–96.9%)	82.5% (75.5%–89.4%)
	ROMA	82.5% (70.7%–94.3%)	83.8% (75.4%–92.2%)	73.3% (60.4%–86.3%)	89.9% (82.7%–97%)	83.3% (76.5%–90.2%)

CI: confidence interval; ROMA: Risk of Ovarian Malignancy Algorithm.

**Table 4:** Diagnostic performance of HE4, CA125, and ROMA for discriminating stage I FIGO malignant adnexal tumors and epithelial ovarian cancer from nonmalignant adnexal tumors.

Groups assessed by diagnostic tests	Diagnostic test	Sensitivity (%) (95% CI)	Specificity (%) (95% CI)	Positive predictive value (%) (95% CI)	Negative predictive value (%) (95% CI)	Diagnostic accuracy (%) (95% CI)
Stage I FIGO malignant adnexal tumor (*n* = 16) versus nonmalignant adnexal tumors (*n* = 252)	HE4	31.3% (8.5%–54%)	92.5% (89.2%–95.7%)	20.8% (4.6%–37.1%)	95.5% (92.9%–98.1%)	88.8% (85%–92.6%)
	CA125	43.8% (19.4%–68.1%)	68.3% (62.5%–74%)	8% (2.3%–13.8%)	95% (91.9%–98.2%)	66.8% (61.2%–72.4%)
	ROMA	43.8% (19.4%–68.1%)	82.5% (77.9%–87.2%)	13.7% (4.3%–23.2%)	95.9% (93.2%–98.5%)	80.2% (75.5%–85%)
Epithelial ovarian cancer (*n* = 46) versus nonmalignant adnexal tumors (*n* = 252)	HE4	76.1% (63.8%–88.4%)	92.5% (89.2%–95.7%)	64.8% (52%–77.6%)	95.5% (92.9%–98.1%)	89.9% (86.5%–93.3%)
	CA125	84.8% (74.4%–95.2%)	68.3% (62.5%–74%)	32.8% (24.3%–41.2%)	96.1% (93.3%–98.9%)	70.8% (65.6%–76%)
	ROMA	82.6% (71.7%–93.6%)	82.5% (77.9%–87.2%)	46.3% (35.5%–57.1%)	96.3% (93.8%–98.8%)	82.6% (78.2%–86.9%)

FIGO: Fédération Internationale de Gynécologie et d'Obstétrique; CI: confidence interval; ROMA: Risk of Ovarian Malignancy Algorithm.

**Table 5:** The AUCs with statistical differences and optimal cut-offs for tumor markers and ROMA in the presurgical differentiation of adnexal tumors in the whole group and premenopausal and postmenopausal subgroups.

Diagnostic test	Whole group			Premenopausal subgroup			Postmenopausal subgroup		
	AUC (95% CI)	*p* value	Optimal cut-off	AUC (95% CI)	*p* value	Optimal cut-off	AUC (95% CI)	*p* value	Optimal cut-off
HE4	0.928 (0.885–0.971)	<0.001	72.4	0.867 (0.739–0.996)	<0.001	54	0.915 (0.857–0.972)	<0.001	92
CA125	0.838 (0.76–0.917)	<0.001	51	0.749 (0.548–0.950)	0.008	42.7	0.891 (0.816–0.966)	<0.001	35
ROMA	0.928 (0.883–0.972)	<0.001	18.2	0.865 (0.729–1.000)	<0.001	8.8	0.917 (0.857–0.977)	<0.001	29.5

AUC: area under the curve; CI: confidence interval; ROMA: Risk of Ovarian Malignancy Algorithm.

**Table 6:** AUCs with statistical differences and optimal cut-offs of tumor markers and ROMA for the differentiation of stage I FIGO malignant adnexal tumors and epithelial ovarian cancer from nonmalignant adnexal tumors.

Diagnostic test	Stage I FIGO malignant adnexal tumors versus nonmalignant adnexal tumors			Epithelial ovarian cancer versus nonmalignant adnexal tumors		
	AUC (95% CI)	*p* value	Optimal cut-off	AUC (95% CI)	*p* value	Optimal cut-off
HE4	0.802 (0.695–0.910)	<0.001	56	0.928 (0.882–0.974)	<0.001	72.1
CA125	0.559 (0.388–0.731)	0.427	—	0.858 (0.781–0.935)	<0.001	54.4
ROMA	0.789 (0.679–0.898)	<0.001	10.5	0.929 (0.882–0.976)	<0.001	18.3

AUC: area under the curve; FIGO: Fédération Internationale de Gynécologie et d'Obstétrique; CI: confidence interval; ROMA: Risk of Ovarian Malignancy Algorithm.

**Table 7:** Results of the Hanley and McNeil test comparing the AUC of the diagnostic tests HE4, CA125, and ROMA for the differentiation of malignant from nonmalignant adnexal tumors.

Compared tests	Malignant versus nonmalignant among the whole group	Malignant versus non-malignant among the premenopausal subgroup	Malignant versus nonmalignant among the postmenopausal subgroup	Stage I FIGO malignant versus nonmalignant adnexal tumors	Epithelial ovarian cancer versus nonmalignant adnexal masses
HE4 versus CA125 (*p* value)	0.043	0.314	0.618	0.017	0.118
HE4 versus ROMA (*p* value)	0.999	0.985	0.965	0.893	0.979
CA125 versus ROMA (*p* value)	0.043	0.324	0.587	0.025	0.112

AUC: area under the curve; FIGO: Fédération Internationale de Gynécologie et d'Obstétrique; ROMA: Risk of Ovarian Malignancy Algorithm.
